# Long Term Prognosis in Closed Head Injury

**Published:** 1967-04

**Authors:** C. Adey


### LONG TERM PROGNOSIS IN CLOSED HEAD INJURY

BY

C. ADEY, Final Medical Student

The purpose of this survey was to investigate the likelihood of return to normal life and employment in patients who had suffered a head injury causing impaired consciousness for over twenty-four hours. In order to confine the study to those in whom unconsciousness could be attributed to direct injury to the brain at the time of the accident, patients with intracranial hæmatoma resulting in cerebral compression, and those with severe multiple injuries were excluded. It was considered important to allow a period of five years for follow-up purposes. On the basis of these criteria one hundred and forty patients admitted to the Neurosurgical Unit at Frenchay Hospital between January 1st 1950 and December 31st 1959 were reviewed. They were classified on the basis of the depth of unconsciousness at the first recorded examination and the time elapsing before full consciousness was regained. Of the one hundred and forty patients whose case records were studied, forty-two died without regaining consciousness, leaving ninety-eight for follow-up. It must be stressed that these cases form a selected group in so far as they were referred to a specialist unit, usually for suspected intracranial complications and cannot be considered typical of closed head injuries in general.

The follow-up was conducted by a postal questionnaire, and replies were received from the patient, a near relative and/or the patient's doctor, in 78 cases. The analysis was based on the information supplied concerning these 78 survivors; some of this may be highly subjective and of doubtful accuracy.

### ANALYTICAL METHOD

The categorisation of patients according to depth and duration of unconsciousness was as follows :—

**Duration :** A. Unconscious for over 24 hrs. but less than 1 week.
B. ,, ,, ,, 1 week ,, ,, ,, 1 month.
C. ,, ,, ,, 1 month.

**Depth :** 1. Responding to voice.
2. ,, ,, pain.
3. No response.

From the questionnaire, each patient was judged as either leading a normal life, or having sustained a persisting disability; particular attention being paid to epilepsy, personality changes, neurological defects, and a change in occupational status.

The influence of the duration and depth of unconsciousness upon each of the persisting disabilities was tested in turn by the method of " exact probability ". In this way any figures that proved to be of statistical significance were discovered. (If the probability that an association was due to chance was less than 1 in 20 it was concluded that the association was likely to be of significance, while if the probability was less than 1 in 100, it was regarded as certainly significant). This preliminary analysis revealed that depth of unconsciousness bore little relation to the final state, and suggested that duration of unconsciousness could profitably be analysed further.

### RESULTS AND DISCUSSION

Table I shows the persisting disabilities attributed to the head injury. Motor deficiencies varied from a limp, or arm weakness, to hemiplegia. Of the patients who had suffered a change in occupational status, 10 had changed their jobs while the other 9 were unemployed. Patients with a psychiatric disorder included those with depression, some with violent tempers, and others with schizophrenia; while the cranial nerves affected were the olfactory, oculomotor, and auditory nerves. The follow-up period was between 5 and

TABLE I

PERSISTING DISABILITIES ATTRIBUTED BY PATIENTS OR THEIR GENERAL PRACTITIONERS TO HEAD INJURY — IN 78 PATIENTS

| Disability | Number with disability |
|---|---|
| Motor deficiency | 20 |
| Decline in occupational status | 19 |
| Psychiatric disorders | 18 |
| Epilepsy | 12 |
| Headache | 10 |
| Memory defects | 9 |
| Cranial nerve lesions | 7 |
| Impaired vision | 5 |
| Impaired concentration | 4 |
| Dysphasia | 2 |
| Cerebrospinal fluid rhinorrhœa | 1 |
| Dizziness | 1 |

15 years, yet in spite of this lapse of time since the injury, 23 of the 78 patients (29.2%) reported some lasting deficit, which was sufficient to prevent them from returning to their normal pre-accident state. Apart from these, a number of other patients had symptoms that were attributed to the injury, but did not impair their ability to lead a normal life. Some complaints were those generally associated with the " post-concussional syndrome ", such as headaches, memory defects, and impaired concentration—these are included in Table I. Irritability and dizziness were not prominent complaints. Following what appeared to be a relatively minor injury, three patients had serious persisting disabilities. One, a male aged 47, became severely demented, and violent to his family; another male aged 51 had headaches accompanied by bouts of screaming, lost his sense of taste and smell, and had cerebrospinal fluid rhinorrhœa; and a third male aged 56 suffered from severe headaches, difficulty in walking, and loss of sight in one eye. This man remained unemployed until his death 4½ years later, from an accident.

From the statistical analysis of the results, it became apparent that the duration of unconsciousness was of prognostic importance, while the depth of unconsciousness on first examination bore no relation to any persisting disability. Tables II to VI show those disabilities that are influenced by a duration of unconsciousness of more than 1 week (persisting neurological disability, post-traumatic epilepsy, and unemployment), or of more than 1 month (ability to lead a normal life, unemployment).

### TABLE II

### INFLUENCE OF DURATION OF UNCONSCIOUSNESS ON THE ABILITY TO LEAD A NORMAL LIFE

| Duration of unconsciousness | Able to lead normal life | Not able to lead normal life |
|---|---|---|
| Less than one month 68 cases | 51 | 17 (25%) |
| More than one month 10 cases | 4 | 6 (60%) |

(P=less than 0.05)

### TABLE III

### INFLUENCE OF DURATION OF UNCONSCIOUSNESS ON PERSISTING (NEUROLOGICAL) DISABILITY

| Duration of unconsciousness | No disability | Neurological disability |
|---|---|---|
| Less than one week 36 cases | 31 | 5 (13%) |
| More than one week 42 cases | 24 | 18 (42%) |

(P=less than 0.01)

### TABLE IV

### INFLUENCE OF DURATION OF UNCONSCIOUSNESS ON DEVELOPING EPILEPSY

| Duration of unconsciousness | Epilepsy | No epilepsy |
|---|---|---|
| Less than one week 36 cases | 2 (5.5%) | 34 |
| More than one week 42 cases | 10 (23%) | 32 |

(P=less than 0.05)

TABLE V

INFLUENCE OF DURATION OF UNCONSCIOUSNESS ON BEING EMPLOYED

| Duration of unconsciousness | Patients unemployed | Patients employed |
|---|---|---|
| Less than one week 36 cases | 1 (2.7%) | 35 |
| More than one week 42 cases | 8 (19%) | 34 |

(P=less than 0.05)

TABLE VI

| | | |
|---|---|---|
| Less than one month 68 cases | 4 (5.8%) | 64 |
| More than one month 10 cases | 5 (50%) | 5 |

(P=less than 0.01)

In this series the incidence of post-traumatic epilepsy (15.4%) is high compared with an overall incidence of 5% for all blunt head injuries (Jennett, 1961). The incidence of a decline in occupational status (24.4%) is lower than the 35% that Miller and Stern (1965) record, but the 15.4% of patients with a disorder of personality compares closely with the figure of 17.4% in Miller's (1966) series.

SUMMARY

From a total of one hundred and forty patients with closed head injuries, forty-two (30%) died without regaining consciousness. Of the remaining ninety-eight, seventy-eight were followed up by questionnaire after a period of five to fifteen years. Of these seventy-eight, twenty-three (29.2%) had persisting disabilities sufficient to prevent a return to normal pre-accident life, and others had persisting symptoms though only nine were unemployed. It is the duration of unconsciousness, rather than its depth on first examination, that was shown to be the statistically significant factor in prognosis.

*Acknowledgements :*

I am grateful to Mr. G. L. Alexander for allowing me to study for this short period in his Unit, during which time I was able to collect material for this study. Also to Mr. A. Hulme and Mr. D. G. Phillips for their advice, and to the secretaries of the Neurosurgical Department at Frenchay Hospital, for their assistance. I wish to thank Miss E. Duncan and Mrs. Morris of the Public Health Department, Bristol University, for their help over statistical problems.

REFERENCES

Jennett (1963) Ann, *Roy Coll. Surg.* **29**, 370.

Miller & Stern (1965), *Lancet*, **i**, 225.

Miller, H. (1966), *Proc. Royal Soc. Med.* **59**, 257.

